# Management of Skin Toxicity Related to the Use of Imatinib Mesylate (STI571, Glivec™) for Advanced Stage Gastrointestinal Stromal Tumours

**DOI:** 10.1080/13577140500349717

**Published:** 2005

**Authors:** Lucy C. Scott, Jeff D. White, Robin Reid, Fiona Cowie

**Affiliations:** 1 Centre for Oncology and Applied Pharmacology University of Glasgow Glasgow Scotland; 2 Beatson Oncology Centre Western Infirmary Glasgow Scotland; 3 Department of Pathology Western Infirmary Glasgow Scotland; 4 Beatson Oncology Centre Western Infirmary Dumbarton Road Glasgow G11 6NT Scotland

## Abstract

Skin toxicity is a common side-effect of treatment with imatinib mesylate (STI571, Glivec™) in advanced gastrointestinal stromal tumours (GIST) and chronic myeloid leukaemia. The optimal duration of treatment with imatinib mesylate in GIST has not yet been established, as durable remissions have been observed in patients. It is, therefore, important to develop strategies to deal with common side-effects of what may be a long-term treatment. Here we report the case of a patient with advanced GIST who developed a cutaneous drug reaction secondary to imatinib mesylate and the various management options that may be employed depending upon the severity of the toxicity. The case and literature are discussed.


*Sarcoma*, September/December 2005; 9(3/4): 157–160

Taylor & Francis

Taylor & Francis Group

CASE REPORT

### Management of skin toxicity related to the use of imatinib mesylate (STI571, Glivec$^{TM}$) for advanced stage gastrointestinal stromal tumours

LUCY C. SCOTT[1], JEFF D. WHITE[2], ROBIN REID[3], & FIONA COWIE[2]

[1]*Centre for Oncology and Applied Pharmacology, University of Glasgow, Glasgow, UK,* [2]*Beatson Oncology Centre, Western Infirmary, Glasgow, UK, and* [3]*Department of Pathology, Western Infirmary, Glasgow, UK*

**Abstract**
Skin toxicity is a common side-effect of treatment with imatinib mesylate (STI571, Glivec$^{TM}$) in advanced gastrointestinal stromal tumours (GIST) and chronic myeloid leukaemia. The optimal duration of treatment with imatinib mesylate in GIST has not yet been established, as durable remissions have been observed in patients. It is, therefore, important to develop strategies to deal with common side-effects of what may be a long-term treatment. Here we report the case of a patient with advanced GIST who developed a cutaneous drug reaction secondary to imatinib mesylate and the various management options that may be employed depending upon the severity of the toxicity. The case and literature are discussed.

**Keywords:** *Gastrointestinal stromal tumours, imatinib mesylate (STI571, Glivec$^{TM}$), skin rashes*

### Case report

A 66-year-old male was originally diagnosed with a gastrointestinal stromal tumour in 2002. The tumour was macroscopically completely excised at laparotomy. He remained well on follow-up for 18 months. He then developed further symptoms. CT scanning confirmed a large mesenteric and omental recurrence. In March 2004 he was, therefore, commenced on the tyrosine kinase inhibitor imatinib mesylate (STI 571, Glivec) 400 mg daily. As his past medical history included hypertension, stroke, type II diabetes mellitus and osteoarthritis, he was on a number of medications including amlodipine 5 mg od, metformin 500 mg bd and lansoprazole 15 mg od, all of which he had been taking for several years. He had no history of drug allergy.

He tolerated the first 8 weeks of treatment with imatinib well, the only toxicities were grade I periorbital oedema and diarrhoea. He was started on loperamide. Follow-up CT scanning at this stage confirmed a good disease response.

At review 4 weeks later the grade I periorbital oedema persisted, but he now had grade II diarrhoea. He had, also, developed a grade I maculopapular rash affecting both forearms, which was felt may be related to either the loperamide or imatinib. As the rash had developed shortly after the loperamide was commenced, the decision was made to discontinue this, and he was changed to codeine phosphate.

However, the rash continued to progress and was exacerbated by exposure to sunlight. When he attended for follow-up at the outpatients' clinic, he was found to have a grade 3 erythematous, maculopapular rash affecting the torso and limbs. The rash was causing significant discomfort and itch. His full blood count at this time showed a moderate eosinophilia of 2.06 (normal range 0.04–0.40). On the advice of the dermatology department he was prescribed Elocon ointment (mometasone furorate) and continued on the same dose of imatinib. A dose reduction of the imatinib was considered at this time, but there were concerns about the risk of tumour flare and, therefore, the decision was made to continue on the current dose of imatinib, but to closely monitor the response to topical steroids.

Correspondence: Dr Jeff White, Beatson Oncology Centre, Western Infirmary, Dumbarton Road, Glasgow G11 6NT, UK. Tel: 44 141 2112000. Fax: 44 141 2116356. E-mail: Jeff.White@NorthGlasgow.Scot.NHS.UK

ISSN 1357–714X print/ISSN 1369–1643 online/05/(03-04)0157-4 © 2005 Taylor & Francis
DOI: 10.1080/13577140500349717

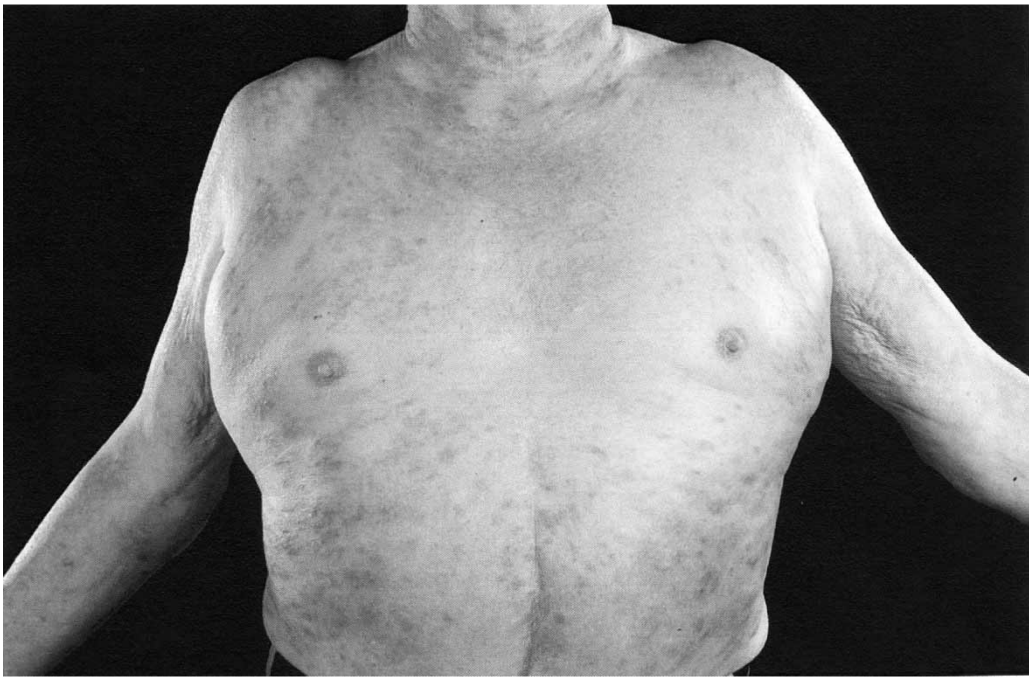

Figure 1. Widespread, erythematous, maculopapular skin rash.

On review at the dermatology clinic 4 days later, his symptoms had improved slightly with decreased erythema and itch. However, on examination he continued to have a widespread excoriated dermatitis affecting the trunk and limbs with areas of sparing in the skin folds and axillae (see Figure 1). As the patient had shown some response to a moderately potent topical steroid, a more potent topical steroid, dermovate (clobetasol propionate) was started as well as an oral antihistamine, chlorpheniramine. He was also encouraged to use plenty of emollients.

Despite applying the more potent topical steroid daily, the rash continued to cause significant symptoms. He was, therefore advised to increase to twice daily applications of dermovate to the trunk and apply eumovate to the face and neck lesions. A skin biopsy was also performed, which showed interphase dermatitis with numerous prominent clusters of colloid bodies in keeping with a drug reaction (Figure 2).

The patient has continued with twice daily applications of the steroid creams, with improvement in the appearance and symptoms from the rash (now grade I) and his eosinophil count is back within the normal range (0.15). He has not required a dose reduction or interruption of imatinib. However, if the rash had failed to respond rapidly to topical steroids a short drug holiday followed by gradual re-introduction of imatinib would have been initiated.

### Discussion

Soft-tissue sarcomas represent 1% of adult malignancies and are derived from mesenchymal tissue. Management options include surgery, radiotherapy and chemotherapy. Gastrointestinal stromal tumours are a sub-type of sarcoma that have a different natural history [1]. They arise predominantly in the stomach (60%) and small intestine (25%), but can arise at a number of other locations including oesophagus, rectum, appendix, gallbladder, pancreas, mesentery, omentum and retroperitoneum. The peak age of occurrence is 60 years (range second to tenth decade). The tumours can be anything between 2 and 30 cm at time of diagnosis and may only be discovered incidentally [2]. Patients with metastatic or locally advanced GIST tumours have limited treatment options, as responses to conventional chemotherapy are very poor with reported objective response rates of <5%.

Imatinib mesylate (STI 571, Glivec) is a small molecule tyrosine kinase inhibitor designed to target c-ABL and BCR-ABL, but is also able to target KIT and platelet-derived growth factor receptor (PDGFR). KIT is highly expressed in GIST[1] and the *KIT* proto-oncogene is often mutated resulting in activation of the kinase. Although KIT expression is not strictly specific, it is highly suggestive of a diagnosis of GIST for sarcomas arising in the digestive tract or abdomen. PDGFR is widely expressed in mesenchymal tissues and the majority of soft-tissue sarcoma sub-types.

A phase I study identified the recommended dose of imatinib as 400 mg twice daily and this dose level had significant activity with objective response rates of 69% and symptomatic benefit in 89% [3]. Phase II studies of imatinib demonstrated that 71% of patients had an objective response and 73%

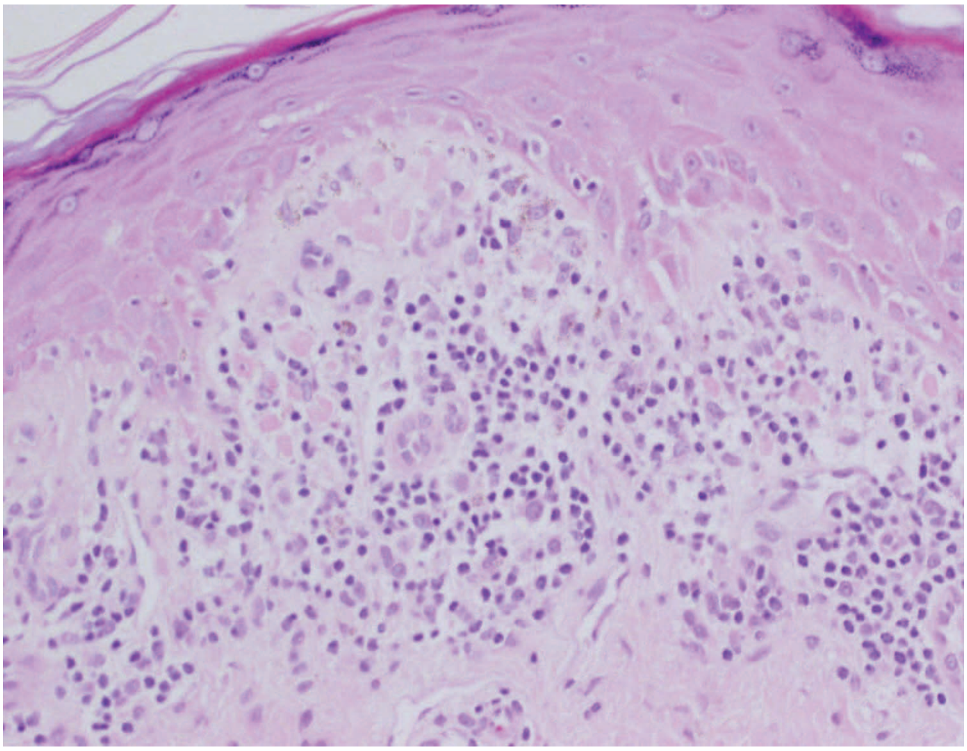

Figure 2. Skin biopsy demonstrating interphase dermatitis.

of patients treated remained progression free at 1 year [4].

The optimal duration of therapy is not yet clear due to the durable remissions observed in patients. As imatinib may be given for long periods it is important to fully characterise the side-effect profile and develop mechanisms for managing toxicities that may arise. Trials of imatinib in GIST to date [4] have shown the most frequent side effects to be anaemia (92%), periorbital oedema (84%), skin rash (69%), fatigue (76%), nausea (57%), granulocytopenia (47%), and diarrhoea (47%). The toxicities are generally mild, grade 1 or 2 severity (NCI-CTC).

Standard management of cutaneous drug reactions usually entails withdrawal of the suspected drug and avoidance of further exposure to this drug in the future. However, in patients with GIST, responses to conventional chemotherapy are very poor, therefore, oncologists (and patients) are keen to avoid permanent withdrawal of imatinib, unless there is no other option.

Skin rashes associated with imatinib usually occur soon after commencing therapy, but may develop many months later. The typical rash is maculopapular and puritic and is distributed predominantly over the forearms, trunk, legs and face [5]. The rash is more likely to occur with higher doses (>600 mg/day) and therefore, may be a pharmacological effect rather than just a hypersensitivity reaction. The majority of these rashes are self-limiting and easily treated with emollients and topical steroids. Usually the patient can continue on the same dose of imatinib. More severe cases may require oral steroids and a dose interruption until the rash has improved to grade I, then a re-challenge with imatinib at a lower dose level (50–100 mg/day) with steroid cover and a gradual dose escalation. The oral steroid dose is usually starting in the range of 0.5–1.0 mg/kg per day of prednisolone or equivalent. Imatinib is predominantly metabolised in the liver by the CYP3A4/5 p450 enzyme system. Glucocorticoids and dexamethasone are potential inducers of this enzyme system and their use could therefore theoretically result in decreased levels of imatinib [5]. However, dose reduction or interruption is usually required in patients with skin toxicity severe enough to require oral steroids. Lansoprazole has been shown *in vitro* to be a potent inhibitor of the cytochrome p450 system [6] and, therefore, it is theoretically possible that this may have resulted in altered pharmacokinetics of imatinib in this patient.

Occasionally the rash can progress to erythroderma which constitutes grade 4 toxicity. This requires immediate and permanent cessation of imatinib and supportive treatment including oral and topical steroids [7]. However, in patients with GIST, there are concerns about sudden withdrawal of imatinib as GIST-disease reactivation after cessation of imatinib therapy has been demonstrated by [$^{18}$F]fluorodeoxyglucose (FDG)-PET scanning. In comparison with CT, FDG-PET reports responses in most patients within 1 week of commencing imatinib, whereas CT scanning may only reveal responses after 2–3 months. Within days of discontinuing imatinib, FDG-PET signals corresponding to the tumour mass become significantly enhanced, suggesting that the tumour cells have reactivated and this may have clinical significance for the patient in terms of symptom control, risk of haemorrhage into the tumour and other complications [8]. However, only a few cases will have skin toxicity severe enough to warrant definitive drug discontinuation.

With regards to the eosinophilia observed in this case, the incidence of eosinophilia in patients on drug therapy is probably less than 0.1%. There is an extensive list of drugs capable of producing skin toxicity and eosinophilia that do not seem to have common chemical or pharmacological properties. In cases where eosinophilia secondary to drug hypersensitivity is suspected on clinical grounds, stopping the drug usually resolves the problem. When an important drug is suspected to be the cause of the hypersensitivity reaction a re-challenge can be attempted. In this situation, the eosinophilic reaction should reappear within 10 days if it is secondary to the suspected drug [9].

### Conclusion

Skin rashes are a common side effect of imatinib. They are, however, usually mild and self-limiting and do not require dose interruption. They generally respond to topical steroids, emollients and antihistamines, but may occasionally require oral steroids. More severe cases may require dose reduction or interruption until the rash has improved to grade I, and re-challenge of imatinib at a lower dose (50–100 mg/day) with steroid cover and gradual escalation in the dose of imatinib. In cases of severe, grade 4 skin rash, re-challenge is not recommended.

In the case reported, the patient developed grade III skin toxicity related to the use of imatinib. This responded well to the use of topical steroids, but, in retrospect, an earlier drug reduction or interruption may have prevented the rash from becoming so severe and consequently requiring potent topical steroids for resolution.

### References

1. Miettinen M, Sarlomo-Rikala M, Lasota J. Gastrointestinal stromal tumors: Recent advances in understanding of their biology. Hum Pathol 1999;30(10):1213–1220.
2. Corless CL, Fletcher JA, Heinrich MC. Biology of gastrointestinal stromal tumors. J Clin Oncol 2004;22(18): 3813–3825.
3. van Oosterom AT, Judson I, Verweij J, Stroobants S, Donato di Paola E, Dimitrijevic S, Martens M, Webb A, Sciot R, Van Glabbeke M, et al. Safety and efficacy of imatinib (STI571) in metastatic gastrointestinal stromal tumours: A phase I study. Lancet 2001;358(9291):1421–1423.
4. Verweij J, van Oosterom A, Blay JY, Judson I, Rodenhuis S, van der Graaf W, Radford J, Le Cesne A, Hogendoorn PC, di Paola ED, et al. Imatinib mesylate (STI-571 Glivec, Gleevec) is an active agent for gastrointestinal stromal tumours, but does not yield responses in other soft-tissue sarcomas that are unselected for a molecular target. Results from an EORTC Soft Tissue and Bone Sarcoma Group phase II study. Eur J Cancer 2003;39(14):2006–2011.
5. Marin D, Marktel S, Bua M, Armstrong L, Goldman JM, Apperley JF, Olavarria E. The use of imatinib (STI571) in chronic myelod leukemia: Some practical considerations. Haematologica 2002;87(9):979–988.
6. Ko JW, Sukhova N, Thacker D, Chen P, Flockhart DA. Evaluation of omeprazole and lansoprazole as inhibitors of cytochrome P450 isoforms. Drug Metab Dispos 1997;25(7):853–862.
7. Sehgal VN, Srivastava G, Sardana K. Erythroderma/exfoliative dermatitis: A synopsis. Int J Dermatol 2004;43(1):39–47.
8. Van Den Abbeele AD, Badawi RD, Manola J, et al. Effects of cessation of imatinib mesylate (IM) therapy in patients (pts) with IM-refractory gastrointestinal stromal tumours (GIST) as visualised by FDG-PET scanning. Proc Am Soc Clin Oncol 2004;22(14S):3012.
9. Brigden ML. A practical workup for eosinophilia. You can investigate the most likely causes right in your office. Postgrad Med 1999;105(3):193–194, 199–202, 207–210.

